# Knowledge, Attitude, and Practices of Dentists in Maharashtra, India, Regarding Sharing Patients’ Data on Social Media: A Questionnaire-Based Study

**DOI:** 10.7759/cureus.88778

**Published:** 2025-07-25

**Authors:** Nilesh V Joshi, Ashwin Jawdekar, Mridula Joshi, Anupa R Shetty, Mirella Vaz, Sanpreet S Sachdev

**Affiliations:** 1 Periodontology, Bharati Vidyapeeth (Deemed to be University) Dental College and Hospital, Navi Mumbai, IND; 2 Pediatric and Preventive Dentistry, Bharati Vidyapeeth (Deemed to be University) Dental College and Hospital, Navi Mumbai, IND; 3 Prosthodontics and Crown &amp; Bridge, Bharati Vidyapeeth (Deemed to be University) Dental College and Hospital, Navi Mumbai, IND; 4 Oral Pathology and Microbiology, Bharati Vidyapeeth (Deemed to be University) Dental College and Hospital, Navi Mumbai, IND

**Keywords:** cross-sectional survey, dentistry, patient photographs, patient records, social media

## Abstract

Background

Social media (SM) has become integral to dental practice, offering opportunities for professional interaction and patient engagement. However, it raises significant ethical concerns, particularly regarding patient confidentiality. The aim of the present study was to gauge the knowledge, attitude, and practice of dental professionals in Maharashtra, India, regarding the sharing of patient data on SM, and to assess the ethical considerations associated with this practice.

Methods

A cross-sectional study was conducted among dental professionals in Maharashtra from October to December 2023. Participants were recruited from six dental institutions and various private clinics. A self-structured questionnaire was validated and distributed via Google Forms (Google LLC, Mountain View, CA, USA). Data were analyzed using MedCalc, version 13.2 (MedCalc Software Ltd, Ostend, Belgium) statistical software, with demographic characteristics and response frequencies summarized in tables and graphs.

Results

Of the 312 participants, 265 completed the survey (response rate: 84.94%). WhatsApp (88.3%) was the most preferred platform, followed by Facebook (54.34%) and Instagram (54.34%). The majority rarely shared patient data on SM. Marketing (63.4%), showcasing treatment results (46.79%), and educating colleagues (46.79%) were primary motivations. While 56.98% agreed that sharing patient data infringes privacy, only 46.04% consistently obtained patient consent. A significant portion (92%) supported establishing guidelines for social media usage.

Conclusion

Dental professionals in Maharashtra frequently use SM for professional purposes, with WhatsApp being the most popular platform. Despite recognizing privacy concerns, there is a lack of consistent patient consent practices. The findings highlight the need for clear guidelines to balance the benefits of social media with ethical obligations to patient confidentiality and professional conduct.

## Introduction

The goal of dental treatment is not only restricted to the restoration of the functioning of the orodental apparatus of an individual, but also to achieve optimal aesthetic outcomes for the entire dentofacial region. In its essence, dentistry comprises the field of medicine in conjunction with art. Dental professionals strive to restore the diseased or missing teeth to a state resembling the natural teeth as closely as possible. It is only natural that one would be tempted to share their achievements with society. In this regard, dentists tend to post pre-treatment and post-treatment photographs and radiographs to showcase the results of the treatment achieved by them, on various social media such as Facebook, Instagram, YouTube, Twitter, and WhatsApp [1]. This action is generally performed owing to various reasons, such as to connect with the other dental fraternity or display their area of expertise in anticipation that they would be consulted for similar cases.

An added advantage of the SM platforms is that the audience is not restricted to only the dental faculty but also extends to include the general population [2]. Gaining their attention would serve to add to the patient flow in their clinical practice. The fact that, in the present day, "branding" plays an important role in commercialization in almost every field is also applicable to the field of dentistry. Thus, an ulterior motive of a professional sharing the photographs of his case on social media could be to enhance their clinical practice [3]. Recently, findings from the #intEHRAct survey conducted across 35 different countries of Europe revealed that about 40% of healthcare professionals share the content of their cases on social media, of which 19% share on a daily frequency [4].

However, while SM can be an extremely useful tool in boosting one’s practice, there are several social, ethical, and legal considerations associated with its use that cannot be overlooked [5]. One must bear in mind the confidentiality of the patient before posting on the SM platform. The professional conduct, etiquette, and ethical regulations of the Indian Medical Council have prohibited medical professionals from self-advertising [6]. They have stated a clause that physicians may not use the identity of the patients in any way to "self-aggrandize" their practice.

However, the earlier regulations were laid down at a time when society was just getting acquainted with the SM, and the field of socialization was undergoing a paradigm shift. The people were not so familiar with the recently introduced gadgets. With time, SM has become an inseparable part of dental practice as well as academics [7]. They are also leveraged to discuss interesting cases and conduct online journal clubs, seminars, and conferences [8]. Their popularity in the present day makes it an excellent opportunity for dental professionals to discuss cases with their colleagues, gain recognition, and boost their consulting practice.

However, dentists need to be wary of the norms for patient privacy protection and appropriate consent before sharing any data on social media. A study in the USA reported that younger dentists are more likely to use social media than older individuals [9]. Only a few studies have assessed and reported the knowledge, attitudes, and practices of dentists regarding the sharing of patients’ data on social media in the Indian context. Much of the health workforce in India, including dentists, is concentrated in the urban areas, particularly the metropolitan cities [10]. It is, thus, essential to understand the knowledge of dental professionals in these areas about sharing their patient data on social media, their opinions, and behaviors related to the matter.

In this context, the present study was conducted aiming to gauge the knowledge, attitude, and practice of the dental professionals in Maharashtra, India, related to sharing the patient’s data on social media. The objective of the study was to determine the patterns of sharing patient data by dental professionals on social media, the reasons for the tendency, and the extent of preservation of ethical integrity in doing so.

## Materials and methods

Study settings

The present cross-sectional study was conducted in accordance with the Declaration of Helsinki, and the protocol was approved by the Institutional Ethical Review Board, Bharati Vidyapeeth (Deemed to be University) Dental College and Hospital, Navi Mumbai (Approval No. BEC433072022, dated August 9, 2023). The participants for the present study were recruited from six dental institutions and several private clinics in the city of Maharashtra over a period of two months, from October to December 2023. Dental clinicians as well as academicians in private clinics, teaching institutes, and consultant practices registered by the State Dental Council were considered eligible for the study. Dentists not currently in practice or not using social media were excluded.

Source of data

A self-structured, 19-item questionnaire was developed to capture dentists’ knowledge, attitudes, and practices (KAP) concerning the online sharing of patient data. It comprised an informed consent question (1 item) followed by five brief sections: (A) demographics (5 items), (B) general social-media use (2 items), (C) posting behavior (3 items), (D) knowledge and purpose of posting (2 items), (E) attitudes and ethical practices (6 items). Response formats included single-choice, multiple-choice, and five-point ordinal scales, and all questions were marked compulsory. The questionnaire was provided to five highly qualified dental professionals for validation and assessment of reliability. Certain minor changes were incorporated as deemed necessary from the initial pilot survey. The questionnaire was then forwarded to a homogeneous sample of 20 middle-aged dentists for testing of external validity. A Cronbach's alpha value of 0.82 indicated good internal consistency and face validity of the questionnaire. The final instrument is reproduced in the Appendices.

Participants were recruited by non‑probability convenience sampling. All faculty and clinicians present in six purposively selected dental institutions, together with private practitioners in their surrounding catchment areas, received the survey link via institutional e‑mail lists and professional WhatsApp groups. Participation was entirely voluntary; no randomization or stratification was attempted. Participants were assured that their identity would not be disclosed and that the data would be used purely for the purpose of the study. Before the commencement of the questionnaire, an informed consent statement was provided to the participants. Participants not consenting to fill out the survey, with incomplete responses, or those who chose to opt out of the survey process were excluded. Because the survey link was circulated through Google Forms via email and messaging apps, coverage was limited to dentists with reliable internet access, introducing a risk of selection bias. To minimize duplicate entries, the form was restricted to one response per Google account, and email ID-based duplicate responses were checked in the response sheet.

Recording and analysis of responses

The data were recorded into an electronic datasheet (Microsoft Excel 2013, Microsoft Corp, Redmond, WA, USA) and checked for errors and discrepancies. Data analysis was performed using IBM SPSS Statistics for Windows, version 23 (IBM Corp., Armonk, N.Y., USA). Descriptive data for the demographics of respondents and frequency of responses are presented as numbers with percentages and summarized in the form of tables/graphs wherever applicable. Because all outcome variables were nominal or ordinal categories, differences in proportions among sub‑groups (e.g., age below/above 40 years, male/female, urban/non‑urban) were evaluated with Pearson’s chi‑square test of independence, the most appropriate test for detecting associations between two categorical variables. For all tests, a p value of 0.05 was considered a threshold for significance.

## Results

A total of N=312 participants were recruited for the present study, of which n=265 completed the survey, thus making the response rate of the present survey 84.94%. The age of the participants ranged from 24 to 81 years, with a mean of 39.48 + 9.14 years. The demographic characteristics of the study population are described in Table 1.

**Table 1:** Demographic characteristics of the study population

Independent variable	Category	No.	% (N=265)
Gender	Male	121	45.66%
	Female	144	54.34%
Region of practice	Mixed	45	16.98%
	Rural	12	4.53%
	Semi-urban	43	16.23%
	Urban	165	62.26%
Type of practice	Private	133	50.19%
	Consultant and teaching	68	25.66%
	Teaching only	20	7.55%
	Any other	44	16.60%
Age group	≤40 years	144	54.33%
	41 to 60 years	115	43.40%
	> 60 years	6	2.27%
Time spent on social media	<30 minutes	85	32.08%
	30 to 59 minutes	73	27.55%
	1 to 2 hours	29	10.94%
	2 to 4 hours	64	24.15%
	>4 hrs	14	5.28%

The most likely social media platform for sharing patient data was reported as WhatsApp by n=234 participants (88.3%), followed by Facebook by n=159 participants (54.34%) and Instagram by n=144 participants (54.34%). Only WhatsApp usage differed significantly by age, being highest in the age group of above 40 years (p = 0.002). No other platform showed significant variation across the compared subgroups. The distribution of responses from the participants of different categories for various preferred social media is summarized in Table 2.

**Table 2:** Most preferred social media platforms as indicated by the respondents (n=265) χ²: Chi square test; df: degrees of freedom Three-category comparison (≤ 40 y, 41–60 y, > 60 y); df = 2 Male vs female; 2 × 2 table with Yates continuity correction; df = 1 Urban vs non-urban (mixed + semi-urban + rural); 2 × 2 table with Yates correction; df = 1 *Includes YouTube, Telegram, X/Twitter, etc.

Platform	Overall n (%)	Age effect χ²(df), p	Gender effect χ²(df), p	Region effect χ²(df), p
WhatsApp	234 (88.3)	12.56 (2), 0.002	0.00 (1), 0.982	1.22 (1), 0.27
Facebook	159 (60.0)	1.09 (2), 0.58	0.32 (1), 0.57	0.09 (1), 0.76
Instagram	144 (54.3)	0.19 (2), 0.91	0.09 (1), 0.77	0.19 (1), 0.66
Other*	22 (8.3)	1.69 (2), 0.43	1.22 (1), 0.27	2.18 (1), 0.14

The majority of the participants reported that they very rarely (n=132, 49.81%) or rarely (n=56, 21.13%) shared patient data on social media. No statistically significant difference (p>0.05) was observed in the frequency of sharing data among different sub-groups of age (p=0.193), gender (p=0.689), practice area (p=0.253), and practice type (p=0.780). About 77% of participants (n=203) never or rarely to sometimes commented on the posts shared by other dentists on social media. A statistically significant difference (p=0.012, <0.05) was noted in the frequency of dentists commenting on the posts shared by other dentists, with a higher number of participants less than 40 years of age commenting ‘sometimes’ and those greater than 60 years of age commenting ‘rarely’ or ‘never’. The results regarding the frequency of sharing patient data are presented in Table 3.

**Table 3:** Frequency of sharing patient data as indicated by the respondents No: Number of responders; χ²: Chi square test

Demographic variable	Category	Very frequently	Frequently	Occasionally	Rarely	Very rarely	χ²	P
Age group	≤ 40 yrs	19 (13.19%)	6 (4.17%)	26 (18.06%)	32 (22.22%)	61 (42.36%)	11.160	0.193
	41–60 yrs	5 (4.35%)	5 (4.35%)	15 (13.04%)	23 (20.00%)	67 (58.26%)		
	> 60 yrs	0 (0.00%)	0 (0.00%)	1 (16.67%)	1 (16.67%)	4 (66.67%)		
Gender	Male	14 (11.57%)	6 (4.96%)	19 (15.70%)	25 (20.66%)	57 (47.11%)	2.257	0.689
	Female	10 (6.94%)	5 (3.47%)	23 (15.97%)	31 (21.53%)	75 (52.08%)		
Practice area	Mixed	4 (8.89%)	0 (0.00%)	6 (13.33%)	6 (13.33%)	29 (64.44%)	14.799	0.253
	Rural	1 (8.33%)	2 (16.67%)	2 (16.67%)	3 (25.00%)	4 (33.33%)		
	Semi-urban	7 (16.28%)	1 (2.33%)	7 (16.28%)	9 (20.93%)	19 (44.19%)		
	Urban	12 (7.27%)	8 (4.85%)	27 (16.36%)	38 (23.03%)	80 (48.48%)		
Practice type	Private	8 (6.02%)	6 (4.51%)	27 (20.30%)	29 (21.80%)	63 (47.37%)	8.062	0.780
	Consultant/institution	8 (11.76%)	3 (4.41%)	9 (13.24%)	14 (20.59%)	34 (50.00%)		
	Institute	3 (15.79%)	1 (5.26%)	2 (10.53%)	3 (15.79%)	10 (52.63%)		
	Other	5 (11.11%)	1 (2.22%)	4 (8.89%)	10 (22.22%)	25 (55.56%)		

Regarding the content shared on social media, clinical photographs were most frequently shared by n=209 (78.87%) respondents, followed by radiographs by n=169 (63.77%), case history by n=80 (30.19%), and other data such as hematological/biopsy/genetic testing reports by n=35 (13.21%) participants. No significant difference (p>0.05) was observed in the content shared by individuals of different categories of age, gender, region, and type of practice (Table 4).

**Table 4:** Frequency of responses regarding the nature of materials shared on the social media by the participants

Demographic variable	Category	Clinical photography	Radiography	Case history	Other
Age group	≤ 40 yrs	118 (81.94%)	94 (65.28%)	44 (30.56%)	21 (14.58%)
	41–60 yrs	87 (75.65%)	71 (61.74%)	33 (28.70%)	13 (11.30%)
	> 60 yrs	4 (66.67%)	4 (66.67%)	3 (50.00%)	1 (16.67%)
Gender	Male	96 (79.34%)	79 (65.29%)	32 (26.45%)	14 (11.57%)
	Female	113 (78.47%)	90 (62.50%)	48 (33.33%)	21 (14.58%)
Practice area	Mixed	33 (73.33%)	26 (57.78%)	13 (28.89%)	7 (15.56%)
	Rural	10 (83.33%)	10 (83.33%)	4 (33.33%)	1 (8.33%)
	Semi-urban	34 (79.07%)	18 (41.86%)	9 (20.93%)	7 (16.28%)
	Urban	132 (80.00%)	115 (69.70%)	54 (32.73%)	20 (12.12%)
Practice type	Private	101 (75.94%)	80 (60.15%)	37 (27.82%)	17 (12.78%)
	Consultant/institution	59 (86.76%)	47 (69.12%)	21 (30.88%)	8 (11.76%)
	Institute	15 (75.00%)	11 (55.00%)	5 (25.00%)	0 (0.00%)
	Other	35 (79.55%)	31 (70.45%)	19 (43.18%)	3 (6.82%)

Four reasons were identified for sharing the patient’s data on social media with similar frequencies, including marketing (n=168, 63.4%), displaying treatment results to others (n=124, 46.79%), educating colleagues (n=117, 46.79%), and seeking a second opinion (n=115, 43.4%). It was observed that marketing was the most common reason stated by a higher number of males, participants with ages less than 60 years, and those practicing in urban/semi-urban areas (Table 5). A greater number of participants (n=8, 66.67%) from the rural areas reported the reason as "educating colleagues." Naturally, there was a significantly greater number of responses in the age group of above 40 years for "educating colleagues" as compared to those below 40 years of age.

**Table 5:** Reasons for sharing patient content on social media as indicated by the respondents CME: continuing medical education; χ²: Chi square test; df: degrees of freedom Age dichotomized as ≤ 40 y (n = 144) vs > 40 y (n = 121) Male (n = 121) vs female (n = 144); Yates continuity correction applied to all 2 × 2 tests Urban (n = 165) vs non-urban (mixed + semi-urban + rural; n = 100) * p < 0.05, statistically significant

Reason	n	% of 265	Age effect χ²(df = 1), p	Gender effect χ²(df = 1), p	Region effect χ²(df = 1), p
Marketing/practice promotion	168	63.4	1.78 (1), 0.182	2.20 (1), 0.138	0.00 (1), 0.979
Showcasing treatment outcomes	124	46.8	0.00 (1), 0.974	5.96 (1), 0.015*	0.11 (1), 0.742
Educating colleagues	117	44.2	10.30 (1), 0.001*	0.58 (1), 0.445	0.73 (1), 0.393
Seeking a second opinion	115	43.4	0.06 (1), 0.802	3.04 (1), 0.081	0.05 (1), 0.818
Other (research, CME, etc.)	14	5.3	—	—	—

About 56.98% (n=151) dentists agreed/ strongly agreed that sharing the patient’s data on social media was an infringement of their privacy, while 11.70% (n=31) expressed disagreement with the statement. A neutral status was maintained by 31.32% (n=83) of the respondents. No significant differences were observed in the levels of agreement or disagreement between the different categories of participants.

Only n=122 (46.04%) of participants always obtained the patient’s consent before sharing their content on social media. The remainder either sometimes (n=52, 19.62%) or never (n=19, 7.17%) acquired the patient’s consent before sharing the content on social media. About 59.5% (n=155) of the participants concealed the patient’s identity before sharing any content on social media, while an additional 9.81% (n=26) did it occasionally. No statistically significant differences (p>0.05) were observed among different categories of participants regarding consent or identity concealment among different subgroups of age (p=0.422), gender (p=0.106), practice area (p=0.151), and practice type (p=0.577). Only n=62 patients (23.4%) faced objection at any point in their practice from a patient to obtain their photograph and post it on social media. This was observed significantly more (p=0.013) in semi-urban and urban areas, while a significantly greater number (p<0.05) of participants from rural regions of practice never faced any such objection.

About 14% of participants responded that they would encourage their colleagues to post patient-related content on social media, while only 7.92% (n=21) of respondents stated that they would discourage their colleagues from posting patient data on social media, and the majority of them (n=174, 65.67%) maintained a neutral status. When asked whether there is a need to establish certain guidelines for posting patient data on social media, about 50.2% (n=133) of participants believed that it was absolutely necessary, while 41.9% (n=111) believed that they could be implemented. Only n=15 participants believed that they were not necessary or did not matter (n=6). Key perceptions regarding privacy, consent, identity concealment, and attitudes toward posting were summarised in Table 6.

**Table 6:** Participant perceptions on privacy, consent practices, identity concealment, and posting behavior (N = 265) χ²: Chi square test; df: degrees of freedom χ² goodness-of-fit tests compare observed response distributions with an equal-proportion null hypothesis for each parameter

Parameter	Response category	n	%	χ²	df	p
Perceived privacy infringement	Agree/strongly agree	151	56.98	81.99	2	< 0.001
	Neutral	83	31.32			
	Disagree/strongly disagree	31	11.70			
Obtains patient consent before posting	Always	122	46.04	84.18	3	< 0.001
	Sometimes	52	19.62			
	Never	19	7.17			
	No response/missing	72	27.17			
Identity concealed before posting	Always	155	59.50	94.51	2	< 0.001
	Occasionally	26	9.81			
	Never	84	31.69			
Patient ever objected to posting	Yes	62	23.40	75.02	1	< 0.001
	No	203	76.60			
Attitude toward colleagues posting cases	Encourage	37	13.96	235.75	3	< 0.001
	Neutral	174	65.66			
	Discourage	21	7.92			
	No response/missing	33	12.45			
Need for formal social-media guidelines	Absolutely necessary	133	50.19	191.92	3	< 0.001
	Could be implemented	111	41.89			
	Not necessary	15	5.66			
	Indifferent	6	2.26			

The attitude toward colleagues posting cases was highly non-uniform (χ² = 235.8, df = 3, p = 8.0 × 10⁻⁵¹). Most respondents were neutral (65.7%), whereas 13.9% encouraged and 7.9% discouraged such postings, and 12.5% offered no opinion. The responses were strongly skewed toward support for regulation. Fully half of the respondents selected “absolutely necessary,” and another 41.9% chose “could be implemented,” leaving only 5.7% who felt guidelines were unnecessary and 2.3% who were indifferent (χ² = 191.9, df = 3, p <0.001).

## Discussion

The present study was conducted with the objective of analyzing the patterns of sharing patient data on social media, the reasons for sharing, and the extent of preservation of ethical integrity by dental professionals. A strong preference was observed for WhatsApp, followed by Facebook and Instagram. This could be attributed to the fact that almost all individuals frequently utilize WhatsApp for instant messaging and broadcasting messages to groups [11]. Other Apps, such as Facebook or Instagram, involve creating and sharing posts for others to view and are relatively less frequently used. An almost equal preference was observed for Facebook and Instagram by the population of the present study, with a slightly greater number of individuals opting to share on Facebook.

The central tendency of the age of the population in the present study was 30 to 40 years. This age group comprises middle-aged adults who are familiar with Facebook, as it was introduced during their youth. Meanwhile, Instagram was introduced relatively later and is a preferred SM for teenagers or young adults. Thus, a higher preference for Instagram may be observed in studies conducted on younger dental professionals. This shift in trend from Facebook to Instagram was also highlighted in a recent online survey of 501 participants by Laor et al. (2022) [12]. Understanding the influence of age on the preference for SM warrants efforts in the form of more research.

About 60% of the participants in the present study reported that their time spent on SM per day was less than an hour. Furthermore, the majority of the participants reported that they rarely ever posted their cases on social media. This finding aligns with an earlier survey-based study that had reported most healthcare professionals are "passive users" of SM, with only 19% sharing content on a daily basis [4]. In general, dental clinicians share only special cases that they need to showcase or challenging cases that they have difficulty diagnosing or deciding on the treatment plan. By displaying optimum results achieved in difficult cases, the dentists can even brand themselves, which was stated as the most common reason for sharing patient data on social media by almost 65% of the participants in the present study. Additionally, senior professionals may share the challenging cases to educate the budding professionals, which was a commonly observed trend among professionals practicing in rural regions in the present study [1,4,8].

Since the patient data is shared for the purpose of showcasing an achievement, educational purposes, or to obtain opinions, it is generally the clinical photographs that are shared [2,3]. Occasionally, the cases have to be backed with radiographs to demonstrate the effect of the pathology on the bone or its improvement by the treatment [13]. Seldom is there a need to share the patient’s case history, which is mostly done in cases of serious pathologies such as diseases or tumors, or if the case is being shared for educational purposes [14,15]. These statements were supported by the findings of the present study, with clinical photographs being shared most commonly, followed by radiographs, and only a fraction of individuals sharing case history.

It would be expected that younger individuals would showcase their results more frequently due to two facts: first, they are more familiar with and spend more time on SM, and second, they are in greater need to market their newly established practice [12]. The findings of this study, however, contrast these statements, wherein, interestingly, it was observed that the frequency of sharing patient information on SM did not differ across individuals of different age groups or areas of practice. Even so, it was found that the younger dentists had a greater propensity to react to or comment on the posts made by other dentists as compared to the older practitioners. This counterintuitive finding may suggest that younger professionals, despite being digitally active, may be more cautious due to increased awareness of data protection issues or professional risks.

Other content that may be shared could be a scientific publication, a case report, or a case spotlight. Certain journals may share cases on a regular basis to promote discussion amongst the medical community or attract audiences [16]. While the contents published in a journal are peer-reviewed and follow stringent privacy policies, the content shared by individuals is not bound by any such norms [17,18]. Some of them may even try to "glorify" their results, or use inappropriate adjectives to "beautify" their case description [19]. In an attempt to aggrandize their skills, the clinicians may end up using certain terms that could be disrespectful or dehumanizing to the patients. This could harm the patient’s reputation, and they could fall victim to discrimination or stigmatization [20]. This also introduces risks of exaggeration, falsification, or the spread of misinformation. This context of misinformation, described as "infodemic," had become a serious issue during the COVID-19 pandemic [21]. Additionally, such exaggeration may not be acceptable to the patient. It has, thus, been recommended that such content must be proofread by the patient as well as a colleague before propagating it to SM [20]. About 23.4% of respondents (n=62) faced objection from a patient to obtaining their photographs and posting them on social media at some point in their practice. The concern was raised more among practitioners in urban areas since the population is generally more educated in these regions.

In this context, about 55% of dental professionals in the present study supported the fact that sharing the patient’s data on social media was an infringement of their privacy. About 92% of the participants showed a positive inclination toward the establishment of certain guidelines for posting patient data on social media. Regulations of the Indian Medical Council 2002 allow clinicians to share content on public health, but with a clause that strongly warns against revealing any personal details or secrets of the patient [6]. The Revised Dentists (Code of Ethics) Regulations, 2014, provided by the Dental Council of India, also prohibit dentists from soliciting patients or boasting of cases, operations, cures, or remedies [22].

Yet, only less than half of the participants obtained consent from the patient before sharing their photographs on the SM. With the advent of newer technologies and the visibility-dependent nature of dental practice, the dependence of dentists on social media has increased over recent years. A change in attitudes related to the aspects of patient privacy has been observed, leading to breaches in doctor-patient confidentiality on a more frequent basis [20]. These observations raise serious ethical concerns that require the immediate attention of the medical and dental councils to lay down solid guidelines to safeguard the interests of the patients.

One limitation of this study is its reliance on self-reported data, which can be subject to response biases such as social desirability bias, where participants may underreport unethical practices or overreport ethical behavior. Additionally, the study's sample is geographically confined to Maharashtra, potentially limiting the generalizability of the findings to other regions with different socio-economic and cultural contexts. The use of an online questionnaire may also exclude those not proficient with digital technology or those who prefer traditional means of communication, potentially skewing the results. Such exclusion may systematically underrepresent older or rural practitioners, potentially skewing the sample toward more digitally savvy individuals. The cross-sectional design captures attitudes and practices at a single point in time, which may not reflect changes over time or account for evolving trends in social media use among dental professionals. The attitudes toward privacy and social media evolve rapidly, especially with technological and regulatory developments (e.g., new data protection laws). Additionally, disseminating the questionnaire online may have excluded dentists with limited internet access, and although one-response restrictions were applied, unrecognized duplicate submissions cannot be wholly ruled out.

## Conclusions

The findings of this study offer important insights into the patterns, motivations, and ethical considerations surrounding the use of social media by dental professionals in Maharashtra. While platforms like WhatsApp, Facebook, and Instagram are widely used for professional purposes, primarily for marketing, education, and showcasing treatment outcomes, significant ethical concerns persist, particularly regarding patient consent and privacy. Less than half of the practitioners consistently obtained informed consent prior to sharing patient data. However, these findings should be interpreted with caution, as the study was limited to internet-using dentists and did not adjust for potential confounders that may influence behavior. Moreover, the cross-sectional nature of the study precludes conclusions about causality or directionality of associations. Despite these limitations, the results underscore the pressing need for formal guidelines and ethical frameworks to support responsible social media use in dental practice, balancing its professional benefits with the imperative to safeguard patient confidentiality and integrity.

## Appendices

Questionnaire

Informed Consent Statement

1.      The present study titled ‘Knowledge, Attitude, and Practices of Dentists in Maharashtra, India, Regarding Sharing Patients’ Data on Social Media: A Questionnaire-Based Study’ to aims to gauge the knowledge, attitude, and practice of dental professionals in Maharashtra, regarding the sharing of patient data on SM, and to assess the ethical considerations associated with this practice. We assure that your identity will be concealed and the data will only be used for the purpose of the study. Do you wish to proceed?

• Yes • No

Section A: Demographic Profile (5 items)

1.      Name: ____________________

2.      Age (years): ______

3.      Gender: • Male • Female • Prefer not to say

4.      Primary practice area: • Urban • Semi-urban • Rural • Mixed

5.      Type of practice (select all that apply): • Private clinic • Consultant • Teaching institute • Teaching + Private

Section B: General Social-Media Use (2 items)

6.      Platforms you currently use (select all that apply): • Facebook • Instagram • WhatsApp • Other: __________

7.      Average daily time spent on social media: • < 30 min • 30 - 59 min • 1 - 2 h • 2 - 4 h • > 4 h

Section C: Posting Behavior (3 items)

8.      How often do you share patients’ data on social media? 1 Never 2 3 4 5 Very frequently

9.      How often do you like or comment on posts shared by other dentists? 1 Never 2 3 4 5 Always

10.   Types of information you usually share (select all that apply): • Clinical photographs • Radiographs • Case history • Other: __________

Section D: Knowledge & Purpose (2 items)

11.   Are you aware of any formal guidelines that protect patient information online? • Yes • No

12.   Your main reasons for sharing patient data (select all that apply): • Seeking a second opinion • Showcasing treatment results • Educating colleagues • Marketing • Other: __________

Section E: Attitudes & Ethical Practices (6 items)

13.   Statement: “Sharing patients’ data on social media infringes on their privacy.” Circle one: 1 Strongly disagree 2 3 4 5 Strongly agree

14.   Do you obtain written or verbal consent before posting patient data? • Always • Sometimes • Never • Not applicable

15.   Do you conceal identifying details (e.g., eyes, name) before posting? • Always • Sometimes • Never • Not applicable

16.   Have patients ever objected to you taking or posting their clinical images? • Always • Sometimes • Never • Not applicable

17.   How likely are you to encourage or discourage colleagues to post patient data? 1 Always discourage 2 3 Neutral 4 5 Always encourage

18.   Need for formal regulations governing online posting of clinical material: • Not necessary • Could be made • Absolutely necessary • Does not matter
